# Political affiliation or need for cognition? It depends on the post: Comparing key factors related to detecting health disinformation in the U.S.

**DOI:** 10.1371/journal.pone.0315259

**Published:** 2025-08-26

**Authors:** Joey F. George

**Affiliations:** Ivy College of Business, Iowa State University, Ames, Iowa, United States of America; National Centre for Scientific Research (CNRS) / Universite Clermont Auvergne / Clermont Auvergne INP, FRANCE

## Abstract

We investigate why people believe disinformation about health-related issues. Acting on disinformation could lead to severe injuries and even death. Five hundred eight American respondents each reviewed 10 different social media posts about health-related topics, and 60% of the posts contained disinformation. They were asked to evaluate the posts for their honesty and explain their decisions. Respondents successfully detected disinformation about 2/3 of the time. Across all participant responses, need for cognition was the only factor important to successful detection of disinformation. When investigating each social media post individually, need for cognition was key for 35% of the posts, while political affiliation was key for 15%. Neither factor was important for the remaining 50% of posts. People with a high need for cognition were adept at detecting online disinformation, but those with conservative political affiliations were not. Those best suited to detecting health-related disinformation either had a high need for cognition or a liberal political affiliation.

## Introduction

We currently find ourselves facing an infodemic – “an overabundance of information – some accurate and some not – that makes it hard for people to find trustworthy sources and reliable guidance when they need it [1].” Widespread access to cell phones with Internet connections and social media have powered the infodemic, as they allow large volumes of information – both accurate and inaccurate – to be disseminated fast and far [2]. The infodemic encompasses misinformation – “false information that may or may not be intentional [3],” and “fake news” – “the presentation of false or misleading information as if it were legitimate journalism [4].” It also includes disinformation - “false, inaccurate, or misleading information designed, presented and promoted to intentionally cause public harm or for profit [5].” While misinformation may be innocent, fake news and disinformation are more sinister, as they are crafted with an intent to mislead.

The infodemic concerns many academic disciplines, including information systems [6], psychology [7], economics [8], and health [9]. The spread of disinformation affects millions of people in their daily lives, from elections to choices about health care. The European Commission [5] stated that disinformation “represents risks for our democratic processes, national security, social fabric, and can undermine trust in the information society and confidence in the digital single market.”

Health care is particularly vulnerable to disinformation. Acting on disinformation can lead to serious injuries and even death. For example, the marketing of chlorine dioxide, a bleach used in water treatment facilities, as a “miracle cure” has led to numerous deaths [10]. Even more alarming, a recent study showed that as many as 17,000 people may have died during the COVID-19 epidemic in the U.S. and Europe because they ingested hydroxychloroquine instead of being vaccinated [11].

There is a large literature on the relationship between disinformation and health. Over 1500 relevant published papers were identified in just four reviews of the literature [12–15]. (See also three special issues on the topic ([16–18]) and a comprehensive review of health communication and misinformation scholarship [19]). Experts [9] have crafted an agenda for additional work in this domain. These include understanding psychological drivers for believing disinformation and assessing the consequences of exposure to it. Scherer and Pennycook [20] state that “it is crucial to understand the underlying factors that lead certain people to believe … false and misleading content online.”

From the beginning, research investigating the credibility of online content has focused on two primary areas: characteristics of the disinformation, and individual differences among its consumers [21,22]. Over the decades, interest has shifted from a broad focus on credibility to a specific focus on disinformation. A key research question that has emerged is the extent to which someone’s political affiliation affects their ability to discern disinformation in the messages they consume. The emerging consensus is that people who profess to follow a conservative political affiliation are more likely to believe disinformation [23]. However, there is evidence that accurate identification of disinformation is more informed by a personality trait – the need for cognition, defined as “the willingness or propensity to think analytically [24].”

The research reported here exposed study participants to social media posts about 10 different health related topics. In addition to deciding if a particular post included disinformation or not, participants also provided the reasoning that justified their decisions. An assessment across all 10 topics demonstrated the importance of the need for cognition in correctly identifying disinformation. However, when investigating assessments of individual social media posts, the role of political affiliation was key in two areas: opinions of political personalities about the COVID-19 vaccine, and governmental warnings about the potential dangers of unproven cures for COVID-19.

## Literature review

Current studies of disinformation in social media have their origin in studies of the Internet’s credibility [2]. These studies considered either the credibility of the Internet in general [22] or the credibility of entire websites [21]. The focus of the research was primarily on characteristics of the Internet/website (e.g., design look, information structure, and information focus [21]) or individual differences among users (demographics such as age and gender, and personality traits such as need for cognition [22]).

With the emergence of social media, the focus of credibility research shifted to online platforms like Facebook and X (formerly known as Twitter). Researchers continued to investigate aspects of online content (e.g., message source [25–28]), and individual differences (e.g., trust in the network, intention to share, need for cognition [29]; emotional state during exposure to social media [30]; and political affiliation [23]). Efforts to understand how users discern the credibility of social media posts have tended to lean more towards individual differences than to aspects of the message or the media.

One of the key individual difference variables, important in both the early credibility studies and in investigations of social media, is the need for cognition. Metzger and Flanagin [22] defined need for cognition as “the degree to which people engage in and enjoy thinking deeply about problems or information and, thus, may be willing to exert effort to critically evaluate information.” This trait is described by Pennycook and Rand [24] as “the willingness or propensity to think analytically” (p. 40), rather than to respond heuristically. Maksl and colleagues referred to it as “a psychological tendency to enjoy thinking [31].” The need for cognition is central to dual processing theories, which argue that humans employ two modes of thinking: one slow and one fast [32], one central and one peripheral [33]. In general, those with a greater need for cognition – the slower, more analytic approach – are better able to discern honesty from dishonesty in online content [24,34]

Traditionally, personality traits have been seen as stable and resistant to change. Current thinking, however, is that personality traits can change over time, especially as people age [35]. Some have argued that need for cognition can be enhanced through such “activities as cooperative learning and argumentation [36].” Cooperative learning and argumentation are both aspects of critical thinking [2,37], and unlike personality traits, critical thinking can be taught. “By cultivating critical thinking skills, individuals can more effectively identify and avoid false, misleading, or manipulative information on social media platforms [2].”

Another key individual difference variable is political affiliation. Studies focusing on fake news have shown that, when compared to those who espouse liberal or centrist views, political conservatives were more likely to: believe fake news stories were true [6,8,38]; believe false headlines [4]; and not recognize fake news [7]. In concert with these findings, a literature review by Baptista and Gradim [23], of papers about North America and Europe, concluded that conservatives were less able to discern fake news than were liberals. Research conducted in three European countries and the U.S. found that “partisanship influences trust far more than ethnic, linguistic, and religious attitudes do…A person’s partisan preference is also a good indicator of who that person is, their values, and what they think” ([39, pp. 350–351]. Hence, political affiliation may be less resistant to change than such personality traits as the need for cognition.

Health studies have investigated both cognitive effort and political affiliation, although separately. Cognitive effort studies, using eye tracking, found some visual stimuli were fixated on longer than others, implying that longer fixations were associated with increased cognitive effort [40,41]. Although the work on political affiliation and disinformation has dealt largely with elections and other political issues, at least two studies investigated political affiliation and reactions to headlines about COVID-19 [7]. These studies found that conservatives were less able to distinguish between real and fake headlines, and that conservatives were likely to agree that COVID-19 was the result of a conspiracy.

Both political affiliation and need for cognition are related to the ability to accurately discern the dishonesty of news. Studies that measured both variables simultaneously [16,34] found that both liberal political affiliation and a greater tendency toward analytical thinking were associated with an increased ability to discern the actual nature of disinformation.

But which factor is dominant? Hence, our research question:

RQ: Does political affiliation or need for cognition play the dominant role in the ability to accurately discern disinformation in social media posts?

## Methods

The study procedures were reviewed and approved by the Iowa State University Institutional Review Board for research involving human subjects (22–290). The study was designed and implemented as an online survey, and it was determined by the IRB to be “exempt from most requirements of the human subject protections regulations.” Consent was obtained from potential participants by presenting them with a written statement about the study on the first screen of the survey. The statement covered issues of confidentiality, use of the data collected, and the possibility that the data might be shared with other researchers. It included the statement “By continuing, you willingly consent to take part in this study.” Only those 18 years old or older could take part in the study. Potential participants were asked to provide their ages, and those under 18 were disqualified and excluded from taking part.

Data were collected by Qualtrics from October 26−31, 2022, from randomly chosen Americans who were members of Qualtrics research panels. An American sample was chosen, as this study was a follow up to two others that used American college students. Also, the American political system is a two-party system. In today’s polarized politics, Democrats are almost all center-left, and Republicans are almost all right or far right. Independents are somewhere in-between, depending on the topic. Republicans constitute 32% of registered voters, Democrats make up 33%, and those with no party affiliation (referred to as Independents) make up the remaining 35%. Into the 1970s, both parties included both conservatives and liberals among their members, but “The conservative movement began to dominate the Republican Party in the 1970s, and then much of the country after 1980 with the presidency of Ronald Reagan. ([42], p. 3).” The domination of conservatives in the Republican party reached its apex with the election of President Donald Trump in 2016. In 2025, Republicans are “overwhelmingly conservative” – 49% say they are conservative, 20% say they are very conservative, and less than 5% identify as liberal. Democrats, on the other hand, identify as very liberal (16%) or liberal (31%); while 45% identify as moderate, and only 6% claim to be conservative [43]. Another survey found Republicans to be even more conservative (77%, with 3% liberal) and Democrats to be even more liberal (58%, with 8% conservative) [44]. The BBC has published a brief primer on the differences between the U.S. political parties [45]. Research showed that their politics affected how Republicans reacted to the COVID-19 pandemic – Republicans reported “lower levels of pandemic threat and riskier pandemic lifestyles” than non-Republicans ([46], p. 9). The overlap, of conservative with Republican and centrist/liberal with Democrats, makes it straightforward to determine ideology by determining political affiliation.

Respondents who failed an attention question were disqualified and excluded from taking part. Screening questions included political affiliation (Democrat, Independent, Republican), household income (<$50,000 USD, $50,000 - $100,000; > $100,000), education (high school to some college; college or advanced degree), and gender (male, female, non-binary or declines to say). The final sample of 508 respondents reflected the responses to these questions: approximately one-third came from each political affiliation, one-third from each income range, and one-half each from the education and gender designations.

Respondents were asked to view 10 social media posts and to determine if each post was honest or dishonest. The posts were chosen from a set of 20 that had been used in previous studies [47,48]. Originally, each respondent was to judge all 20 posts, but Qualtrics consultants thought the task would then be too much work, so the set of posts was split in two. Accordingly, there were two separate but parallel data collection efforts, each utilizing 10 unique posts. The first version of the instrument had 253 respondents; the second had 255. The first version of the instrument took on average 10 minutes to complete; the second version took 11 minutes.

The original set of 20 social media posts included two posts for each of 10 topics, so dividing the posts across two versions of the instrument was straightforward. Each version contained one post about each topic. Sixty percent of the posts were dishonest (Appendix 1). Both posts about COVID-19 vaccines and both posts about diabetes cures were dishonest; for every other topic, there was one honest and one dishonest post. Each version of the instrument had the same proportion of honest and dishonest posts.

After viewing a post and recording whether it was honest or dishonest, each respondent chose up to eight reasons to justify their choice. The reasons provided were derived from the results of two previous studies [47,48]. If a respondent decided a post was honest, the survey instrument branched to a set of reasons for deciding the post was honest. If they thought it was dishonest, the instrument branched to a different set of reasons (Table 1). Respondents could also provide additional reasons in a text box.

**Table 1:** Reasons for deciding a post is honest or dishonest.

Reasons for Deciding a Post is Honest	
Item	Text
1	It’s from a reliable source.
2	I’m familiar with the subject matter.
3	It looks promising or possible.
4	The photo looks real and true.
5	It has a professional look.
6	It provides a practical explanation.
7	It provided links to other information.
8	It’s a verified Twitter account.
**Reasons for Deciding a Post is Dishonest**	
Item	**Text**
1	The post looks fake.
2	The claims made are unsubstantiated.
3	The post looks unprofessional.
4	This is an obvious falsehood.
5	It’s a biased ad.
6	There are no credentials provided.
7	The post is based on opinion.
8	It’s from a random person.

Need for cognition was measured with a subscale of the News Media Literacy scale [31]: Questions about Automatic vs Mindful Thought Processing. There were five items: (1) I don’t like to have to do a lot of thinking; (2) I try to avoid situations that require thinking in depth about something: (3) I prefer to do something that challenges my thinking abilities rather than something that requires little thought; (4) I prefer complex to simple problems; (5) Thinking hard and for a long time about something gives me little satisfaction. The subscale was measured on a 5-point Likert scale, from 1 for ‘strongly agree’ to 5 for ‘strongly disagree.’ Items 1, 2 and 5 were reversed coded and had to be recoded for analysis.

Respondents were then asked for additional demographic information, including age, ethnicity, and highest educational level of a parent (Table 2).

**Table 2:** Descriptive data for each sample.

	Version 1	Version 2
Democrat	34%	33.5%
Independent	33%	33%
Republican	33%	33.5%
Low income	34%	35%
Middle income	36%	35%
High income	30%	30%
Gender range	49% M; 49% F; 1% other	49% M; 51% F; < 1% other
Education range	49% HS; 48% C; 3% unsure	49% HS; 47% C; 4% unsure
Age range	19–92 (42% over 60)	18–91 (41% over 60)
Need for cognition	47% high need	48% high need
Ethnicity	83% W; 8% B; 3% AAPI	86% W; 7% B; 4% AAPI
Parental education	47% no degree; 53% degree	42% no degree; 58% degree

Legend: Gender: M – male, F – female; Education: HS – high school, C – college; Ethnicity: W – White, B – Black, AAPI – Asian American & Pacific Islanders.

## Results

Respondents to Version 1 (V1) of the instrument were 65% successful at identifying the actual nature of the posts they were exposed to. Respondents to Version 2 (V2) were 67% successful (Table 3). When the data were pooled, the overall success rate was 66.2%. Parental education was not related to detection success (V1: r = −.069, 2-tailed p = .276; V2: r = −.085, 2-tailed p = .178). Also, there was no relationship between participant education level and political ideology (pooled data: r = −.703, 2-tailed p = .103).

**Table 3:** Truth success rates by post by instrument version.

Version 1				
	Order	Source	Veracity	% Correct
COVID-19 vaccines: Ben Carson’s experiment on children	1	Newsweek	Dishonest	45%
Weight loss regimes: FDA semaglutide test results	2	Twitter	Honest	52%
Common cold cure: Brandy cocktail	3	Pinterest	Dishonest	76%
Ivermectin: FDA ivermectin warning	4	Twitter	Honest	66%
Antibiotics for viruses: Do not take antibiotics for viruses	5	Twitter	Honest	86%
Chlorine dioxide: Should be used as prophylactic and cure	6	scribd.com	Dishonest	75%
MMR vaccine: MMR vaccine is safe, measles is deadly	7	Twitter	Honest	54%
Muscle building supplements: Build muscle fast	8	truthinadvertising.com	Dishonest	76%
Diabetes cure: OTC Berry Gen to treat and cure diabetes	9	Amazon.com ad	Dishonest	50%
COVID-19 cocktail: Cure COVID-19 with drug cocktail	10	Twitter	Dishonest	71%
**Version 2**				
	**Order**	**Source**	**Veracity**	**% Correct**
COVID-19 vaccines: Luciferase implies Satan (Newsmax)	1	Twitter	Dishonest	80%
Weight loss regimes: Intermittent fasting works	2	Twitter	Dishonest	44%
Common cold cure: Nyquil	3	Amazon.com ad	Honest	86%
Ivermectin: Take ivermectin to cure COVID-19	5	Blog post: No longer available on the web	Dishonest	73%
Antibiotics for viruses: Take antibiotics to cure COVID-19	6	Twitter	Dishonest	84%
Chlorine dioxide: FDA chlorine dioxide warning	7	Twitter	Honest	83%
MMR vaccine: RFK and anti-MMR propaganda in Samoa	8	Twitter	Dishonest	58%
Muscle building supplements: Harvard Public Health warning	9	Twitter	Honest	64%
Diabetes cure: OTC Lysulin to treat and cure diabetes	10	Amazon.com ad	Dishonest	31%
COVID-19 cocktail: FDA hydroxychloroquine warning	4	Twitter	Honest	72%

Note: Semaglutide is a drug developed by Novo Nordisk, which is sold under the names Ozempic, Wegovy, and Rybelsus. It is used to treat diabetes type 2 and for weight management.

If the data are weighted for three demographic variables, based on the differences between sample data collected and 2022 U.S. census data, the success rates vary. Given that the sample was designed to be half male and half female, the weighted success rate was the same as the unweighted one: 66.2%. Data on education were collected to be equal portions of those with a 4-year college degree or better and those whose education stopped short of earning a 4-year degree. The latter represented 65.2% of the U.S. population in 2022, so the weighted success rate was 65.7%. Data were also collected for age and were grouped into four categories used by the U.S. census. The weighted success rate for age was 65.5%. Across these three demographic variables, success rates were reasonably high. The lowest success rate was 62.2% for those aged 21–44, while the highest rate was 69.1% for those with a 4-year degree. (See Appendix 2 for more details about success rates by variable category and how weights were calculated and applied.) In general, the combined sample closely approximates the U.S. population in 2022, when the data were collected.

The order of exposure was not identical for each version of the instrument. Respondents for V2 saw the post about COVID-19 cocktails fourth. The remaining posts were then presented in the same order as in V1. This allowed V2 respondents to have two honest posts in a row, for posts 3 and 4, just as V1 respondents saw two honest posts in a row in posts 4 and 5.

Several posts had success rates under 60% for both versions of the instrument: 1) weight loss regimes, 2) the MMR vaccine, and 3) diabetes cures. Respondents for V1 also had a low success rate for the COVID-19 vaccine post, at 45% correct. There were high success rates for other topics: 1) common cold cure, at 76% or better, 2) antibiotics for viruses, at 84% or better, 3) chlorine dioxide, at 75% or better, and 4) COVID-19 drug cocktails, at 71% or better.

Two different statistical tests were needed to test the RQ: an overall test using general estimating equations (GEE), and a separate test for each social media post using the generalized linear model, run in SPSS 27. The former was used due to the repeated measures nature of the data and because the dependent variable was binary (successful or unsuccessful detection). The latter method was used due to the binary nature of the dependent variable.

The five items in the need for cognition scale were analyzed via exploratory factor analysis. The data from both versions of the survey instrument were pooled for this process. The details are contained in Appendix 3. Three of the five items loaded on one sub-scale, while the other two loaded on a second. The items in the first sub-scale had high loadings on the factor, and the scale had high values for measures of construct reliability, Cronbach’s alpha, and average variance extracted. However, as the model with three items was saturated, it was not possible to calculate the goodness of fit statistics that would normally result from using structural equation modeling for a confirmatory factor analysis test. To improve the scale, one item (MAC5R) was removed as it had a factor loading of.679, below the recommended threshold of.7. The new two-item scale had higher values for construct reliability, Cronbach’s alpha, and average variance extracted than the three-item scale. Need for cognition scale values were then calculated by averaging values for the two items, providing a continuous variable. For use in the GEE procedure, it was converted to a factor with two values, high need and low need, based on the mean of the scale. Descriptive statistics for need for cognition and success at disinformation detection are contained in Table 4.

**Table 4:** Descriptive statistics.

Variable	N	Min	Max	Mean	Std Dev	Scale
Correct Judgment	2550	1	2	1.33	.469	1 = correct; 2 = incorrect
Need for Cognition	253	1	2	1.50	.500	1 = high need; 2 = low need

A regression was run to determine the effect of need for cognition and political affiliation on correctly identifying the veracity of the social media posts. The dependent variable was the total number of posts that were correctly identified, and since the measure was across posts for each participant and not based on their evaluations of individual posts, the data could be pooled. Both independent variables were found to affect the number of posts that were correctly identified (Table 5), although the effect of need for cognition was stronger.

**Table 5:** Effect of need for cognition and political affiliation on disinformation detection.

Independent Variable	Unstandardized Coefficient	Standard Errors	P-value
(Constant)	8.430	.259	<.001
Need for Cognition	−.538	−.316	<.001
Political Affiliation	−.244	−.117	.006

R^2 ^= .114.

F-ratio = 32.228 (p < .001).

N = 505.

Cohen’s f = 0.359 (large effect size).

Note: The dependent variable is measured as the total number of posts correctly identified (honest or dishonest) by each participant.

To determine the effects of political affiliation and need for cognition on detection success across the 10 media posts in each version of the instrument, two GEE tests were run, one for each version.. For both versions, political affiliation had no effect on detection success, but need for cognition was statistically significant (V1: Wald chi-square = 15.01, df = 1, p < .001; V2: Wald chi-square = 29.78, df = 1, p < .001). Respondents with a higher need for cognition were more successful at detecting disinformation, compared to those with a lower need (V1: Higher need & success: 69%, Lower need & success: 61%; V2: Higher need & success: 72%, Lower need & success: 62%).

To determine if this relationship held for individual posts, a GLM test was run for each post in each version of the instrument (Table 6). For V1, five posts showed statistically significant relationships between need for cognition or political affiliation on detection success: 1) anti COVID-19 vaccines (quoting prominent Republican politician Ben Carson), 2) a cocktail for curing the common cold, 3) an ivermectin warning from the FDA, 4) an ad for Berry Gen, purported to treat and cure diabetes, and 5) a pro cocktail post of drugs for treating COVID-19 (Kelly Victory). For posts one and three, political affiliation was the significant factor related to detection success; for the other posts, need for cognition was the significant factor. Only the post about ivermectin was honest. The posts were presented first, third, fourth, ninth and tenth.

**Table 6:** Social media posts where detection success was significantly influenced by either need for cognition or political affiliation.

Social Media Post	Omnibus Test	Key Factor	Test of Significance	Cramer’s V	Percent Correct
Anti COVID-19 vaccines (F)	24.58, df = 3, p < .001	Politics	28.5, df = 2, p < .001	.335*	D 51%, I 60%, R 24% (D = I > R)
Common cold cocktail (F)	9.57, df = 3, p = .023	Need for cognition	7.0, df = 1, p = .008	.167	HC 83%, LC 69%
Ivermectin warning (T)	9.25, df = 3, p = .026	Politics	8.61, df = 2, p = .014	.185	D 78%, I 59%, R 60% (D > I = R)
Berry Gen (F)	14.97, df = 3, p = .002	Need for cognition	14.13, df = 1, p = .001	.236	HC 62%, LC 38%
Pro COVID-19 cocktail (F)	12.67, df = 3, p = .005	Need for cognition	11.79, df = 1, p < .001	.216	HC 81%, LC 61%
Luciferase (F)	8.18, df = 3, p = .042	Need for cognition	5.60, df = 1, p = .018	.149	HC 86%, LC 74%
Anti hydroxychloroquine (T)	13.48, df = 3, p = .004	Politics	12.57, df = 2, p < .002	.222	D 84%, I 74%, R 59% (D = I > R)
Pro ivermectin (F)	22.81, df = 3, p < .001	Need for cognition	17.94, df = 1, p < .001	.266	HC 85%, LC 61%
Pro antibiotics for COVID-19 (F)	25.86, df = 3, p < .001	Need for cognition	17.93, df = 1, p < .001	.266	HC 94%, LC 73%
Anti MMR vaccine (F)	16.02, df = 3, p = .001	Need for cognition	14.92, df = 1, p < .001	.242	HC 69%, LC 45%

Legend: Political affiliation: D – Democrat, I – Independent, R – Republican; Need for cognition: HC – High need, LC – Low need.

* Considered a medium effect; all other effects are considered small [49].

For V2, five posts also showed statistically significant relationships: 1) a post that claimed that COVID-19 vaccines contained an agent called Luciferase that allowed people to be tracked, 2) a hydroxychloroquine warning from the FDA (COVID-19 cocktail), 3) a pro ivermectin post (unsigned), 4) a pro antibiotics for COVID-19 post (Yolanda Miranda), and 5) an anti MMR vaccine post (citing Robert Kennedy Jr. during the measles outbreak in Samoa in 2019). Only the post on hydroxychloroquine was honest. This was the only post where political affiliation was the significant factor related to detection success in V2. These posts were presented first, fourth, fifth, sixth, and eighth. (See Appendix 4.)

Across both versions of the instrument, the topics where need for cognition was the key factor were the common cold cocktail, Berry Gen, pro-COVID-19 cocktail, Luciferase, pro-ivermectin, pro-antibiotics to treat COVID-19, and anti-MMR vaccine. All seven of these posts were dishonest, and participants with a high need for cognition were able to discern that successfully. The three posts where political affiliation was key to discernment were anti-COVID-19 vaccines (quoting Ben Carson), an FDA warning about ivermectin, and an FDA warning about hydroxychloroquine. Both posts from the FDA were honest; the post quoting Ben Carson was not. Republicans were more likely to believe the Ben Carson post, Republicans and Independents were least likely to believe the ivermectin warning, and Republicans were least likely to believe the hydroxychloroquine warning. Alternatively, Democrats were successful at discerning the actual nature of these three posts.

Those who believed that the dishonest Ben Carson post was honest cited Carson’s reliability as a source, that the statement – that giving the COVID-19 vaccine to children was a vast governmental experiment – looked promising or possible, and that the photo of Carson in the post looked real. It seems the political nature of the post was related to both Carson and the COVID-19 vaccine, which was controversial among conservatives, making it seem honest to them but not to Democrats or Independents.

Those who believed that the honest FDA warnings about ivermectin and hydroxychloroquine were instead dishonest reported that the warnings looked false and made unsubstantiated claims. The government warnings were about two potential cures for COVID-19 that were favorites of conservatives. It would be expected that people who did not trust the U.S. federal government about recommended treatments for COVID-19 would suspect its motives in warning people away from these alternative cures.

The seven dishonest posts, where need for cognition was the key factor in discerning disinformation, were all successfully evaluated by those with a higher need for cognition. The three most popular reasons provided were unsubstantiated claims (15% or more of the total), the source was a random person (14% or more), and the source provided no credentials (13% or more). However, the salience of reasons cited varied across posts.

## Discussion

Our research question was:

RQ: Does political affiliation or need for cognition play the dominant role in the ability to accurately discern disinformation in social media posts?

Overall, respondents were moderately successful at detecting disinformation, at 65% (V1) or better (67% for V2). While these success rates are not high (a D+ grade), they are better than the detection success rate reported in the deception literature of 54% [50].

Respondents tended to believe disinformation about multiple health-related topics. A majority of respondents believed disinformation about health care regimes based on intermittent fasting (56%) and about over the counter cures for diabetes type 2 (69% and 50%). Forty-two percent believed disinformation about the MMR vaccine. A majority of V1 respondents were unable to detect the disinformation in a post about the COVID-19 vaccine voiced by Ben Carson (55%).

For both versions of the survey instrument, considering overall performance for all respondents on all items, need for cognition was the key factor in successful detection. However, considering performance on a question-by-question basis, neither political affiliation nor need for cognition were factors in half of the posts. For the remaining posts, need for cognition was the key factor in seven cases, with political affiliation being a key factor in three. Where need for cognition was key, those with a high need for cognition were better at discerning disinformation than were those with a low need. Where political affiliation was key, Democrats (liberals) were always better at discerning disinformation than were Republicans (conservatives).

Where political affiliation was the key factor, the most cited reasons for deciding a dishonest post was honest were reliable source, the possible or promising look of the post, and the genuine look of the photo. The two most cited reasons for deciding an honest post was dishonest were that the post looked false and the claims it made were unsubstantiated. Where need for cognition was key, the most cited reasons for correctly deciding the post was dishonest were that the claims it made were unsubstantiated, it originated from a random person, and a lack of author credentials.

The importance of need for cognition to successfully detect disinformation about health is supported by related research. Pennycook and Rand [24], for example, found consistent evidence that analytical thinking played a key role in how people judge the veracity of fake news. Other research supports this view [34,51,52], although a 2023 study found that a need for cognition was associated with a susceptibility to misinformation [53]). The importance of political affiliation was also demonstrated in our findings, in line with findings from previous work: [4,6–8,23,38,51,54]. As important as political affiliation turned out to be in this study, it played less of a role overall than need for cognition. The focus of this paper was a contrast between the roles played by need for cognition and political affiliation, but it is important to note that other factors play a role: educational attainment, health literacy, attitudes toward alternative medicine, trust in the health care system [55], source credibility [54], reliance on social media, and reliance on alternative health media [53].

The results of the study provide insight into the role that context plays in the evaluation of the veracity of social media posts. For both posts about chlorine dioxide as a prophylactic and cure, detection success rates were quite high – 75% recognized the pro-CD post as false, while 83% correctly judged the FDA warning about CD as true. For both posts about dieting, on the other hand, success rates were relatively poor – 52% correctly judged the post about semaglutide as true, while 44% correctly judged the post about intermittent fasting as false. Regarding muscle building supplements, 76% correctly identified the ad for Xtreme Nitro as false, and 64% correctly identified the post from Harvard Public Health, which warned against such supplements, as true. In all three of these cases, need for cognition and political affiliation were not important to participants’ evaluations. As a group, they largely understood the dangers of CD and muscle building supplements, while they were somewhat equivocal on the veracity of either claim about dieting. The three posts where political affiliation was central to how participants made their judgments were all about politically charged topics related to COVID-19. The post about Ben Carson involved a political official from the first Trump administration, who was denigrating COVID-19 vaccines. The other two posts were both issued by the FDA, warning against the use of particular drugs for treating COVID-19. These drugs, ivermectin and hydroxychloroquine, had been heavily promoted by conservatives as cures for the virus. That conservatives would believe Ben Carson and distrust the FDA regarding these two particular drugs is not surprising. Carson’s credibility as a source was no doubt important to them, but they questioned the FDA’s credibility regarding these drugs. However, posts issued by the FDA about CD and semaglutide were both recognized as honest, regardless of participant political affiliation. It seems, then, that the idea of source credibility is nuanced when considering the detection of disinformation [54]. It is important to consider both the source of the post and the topic discussed. Here even generally reliable sources (e.g., the FDA), that were seen as trustworthy on some topics, were not trusted when their views ran counter to other factors, such as strongly held political views.

### Implications, limitations and future research

This study has implications for both research and practice. For 50% of the posts used in this study, investigated individually, neither need for cognition nor political affiliation was related to successfully detecting the actual nature of the post. For 35% of the posts, those with a high need for cognition successfully detected disinformation, and for 15%, a conservative political outlook resulted in incorrect assessments. For successful detection of disinformation, either a high need for cognition or a left-leaning political outlook would therefore be preferable. The best detector of disinformation would have one or both of these characteristics. Alternatively, training programs could be developed to increase people’s need for cognition or to alter their political affiliations. Given current thinking that personality traits can change, and given the hardening of partisanship around the world, a focus on enhancing need for cognition should be more successful than attempting to alter political beliefs.

As mentioned previously, critical thinking skills can be viewed as important tools for evaluating the veracity of disinformation in social media. There is some evidence that critical thinking is related to need for cognition [56,57], although it is difficult to determine whether those with a need for cognition seek out critical thinking skills or those who develop such skills enhance their need for cognition. There is some evidence that critical thinking skills are associated with successful detection of disinformation [37]. There is also evidence that a lack of critical thinking skills, such as reliance on religious fundamentalism [58], or partisan and political biases [2], impede the ability to successfully detect disinformation. Other research [59] found that individuals with high levels of fluid intelligence – “the ability for inductive and deductive reasoning as well as the ability for quantitative (mathematical) reasoning (p. 3)” – were better able to cope with misinformation than those who lacked it.

Scholars have called for increased use for artificial intelligence (AI) in detecting disinformation, whether in the form of machine learning [60], government supplied AI-based assistants that help citizens use the Socratic method to determine the veracity of social media content [61], or companies that use information advantage and machine learning platforms to work with government and industry [62]. There is now some evidence for the success of an AI approach to address conspiracy theories and those who believe them. In a study using large language models-based GPT to generate counter arguments to conspiracy theories, participant belief in a conspiracy theory was reduced by 20%. [63]. Further, this effect continued for at least the two-month duration of the study. Another approach would be to rely on proven strategies for arousing suspicion about the veracity of social media. These include teaching people to consider the risk in spreading disinformation; question the source of the post; and investigate the particular issue [64].

All research has limitations. This study had a relatively large sample, but it was limited to American adults who belonged to a Qualtrics panel. Further, data were collected in late 2022, when the COVID-19 pandemic was ending, and people were putting it behind them. Future similar studies would benefit from different samples, with data collected from respondents representing other countries, languages, and cultures. Data could also be collected from a new U.S. sample, removed several years from the pandemic’s end. Many of the anxieties associated with COVID-19, and how today’s respondents view older stimuli would be different. Future studies would also benefit from developing a new set of stimuli on health issues, given the number of posts in this study about COVID-19 and drawn from Twitter (now X). As X, Twitter has evolved considerably and may well feature more disinformation than the original Twitter did. Other newer social media platforms, such as Meta’s Threads, could be considered instead. Still another approach, which would be aimed at teasing out the role of political affiliation and partisanship, would involve building a set of stimuli that mentioned party names and positions explicitly [65,66]. Posts that were pro-in-party (which explicitly name the party currently in power) and those that were pro-out-party (which explicitly name the party currently out of power) could be compared to help make the role of partisanship in detecting health disinformation easier to discern. Finally, recruiting adults suffering from chronic diseases for a study like this one would provide a different perspective on detecting disinformation in health-related social media posts.

## Conclusion

The infodemic has made it difficult for people to obtain accurate and reliable information online. Disinformation spreads ever faster and farther. We have demonstrated that successful detection of false information is possible, although most people would barely achieve a passing grade for their efforts. And while people with a high need for cognition are adept at detecting online disinformation, those with conservative political affiliations are not. Given the nature of the infodemic, there continues to be a need for additional research, about disinformation and health-related issues that affect people’s daily lives.

## Supporting information

S1 Appendix Documentary evidence of disinformation in posts considered false. (DOCX)

S2 Appendix Tables for weighting samples. (DOCX)

S3 Appendix Factor analysis results. (DOCX)

S4 Appendix Statistical test results for non-significant posts. (DOCX)
