# Supplementary material for: Political affiliation or need for cognition? It depends on the post: Comparing key factors related to detecting health disinformation in the U.S
Source: PLoS One. 2025 Aug 26;20(8):e0315259. doi: 10.1371/journal.pone.0315259 (PMC12380328; doi:10.1371/journal.pone.0315259)
Supplement: S2 Appendix — (DOCX) [file pone.0315259.s002.docx]

**Appendix 2: Tables for Weighting Samples**

Two samples were pooled for these analyses. N = 508

Percent correct in their assessments of disinformation content: Unweighted sample: 66.2%

|  | Correct | Incorrect |
| --- | --- | --- |
| Gender |  |  |
| Male | 65.7% | 34.3% |
| Female | 66.7% | 33.3% |
| Education |  |  |
| Less than 4-year college degree | 64.0% | 36.0% |
| 4-year college degree or higher | 69.1% | 30.9% |
| Don’t know | 57.1% | 42.9% |
| Age |  |  |
| 18-29 | 75.0% | 25.0% |
| 21-44 | 62.2% | 37.8% |
| 45-64 | 67.0% | 33.0% |
| 65+ | 68.4% | 31.6% |

Note: Those who answered “don’t know” about their educational level were all young respondents, currently being educated. They did not know what their highest educational attainment would be.

Weighted results:

US numbers come from 2022 US census data, 18 or older; age categories used come from US census practice. Weights are calculated by dividing the population proportion by the completed survey proportion.

|  | US census | Combined surveys | Weights |
| --- | --- | --- | --- |
| Gender |  |  |  |
| Male | 48.8% | 49.2% | 0.99 |
| Female | 51.2% | 50.0% | 1.02 |
| Education |  |  |  |
| Less than 4-year college degree | 65.2% | 49% | 1.33 |
| 4-year college degree or higher | 34.8% | 47.6% | 0.73 |
| Don’t know |  | 3.3% | ----- |
| Age |  |  |  |
| 18-29 | 4.7% | 2.4% | 1.96 |
| 21-44 | 41.1% | 29.9% | 1.37 |
| 45-64 | 32.3% | 34.6% | 0.93 |
| 65+ | 22.0% | 33.1% | 0.66 |

Applying Weights to Adjust Overall Detection Success Rates:

|  | Count | Weights | Adjusted Count | Proportion Correct | Adjusted Totals | Weighted Proportion Correct | Unweighted Proportion Correct | Delta |
| --- | --- | --- | --- | --- | --- | --- | --- | --- |
| Gender | | | | | | | | |
| Male | 2500 | 0.99 | 2475 | 0.657 | 1626 | 66.2% | 66.2% | 0.0% |
| Female | 2540 | 1.02 | 2591 | 0.667 | 1728 |  |  |  |
| Total | 5040 |  | 5066 |  | 3354 |  |  |  |
| Education | | | | | | | | |
| < College | 2490 | 1.33 | 3312 | 0.64 | 2119 | 65.7% | 66.5% | 0.8% |
| > College | 2420 | 0.73 | 1767 | 0.69 | 1219 |  |  |  |
| Total | 4910 |  | 5078 |  | 3338 |  |  |  |
| Age Range | | | | | | | | |
| 18-20 | 120 | 1.96 | 235 | 0.75 | 176 | 65.5% | 66.2% | 0.7% |
| 21-44 | 1520 | 1.37 | 2082 | 0.62 | 1291 |  |  |  |
| 45-64 | 1760 | 0.93 | 1637 | 0.67 | 1097 |  |  |  |
| 65+ | 1680 | 0.66 | 1109 | 0.68 | 754 |  |  |  |
| Total | 5080 |  | 5063 |  | 3318 |  |  |  |
