# Supplementary material for: Political affiliation or need for cognition? It depends on the post: Comparing key factors related to detecting health disinformation in the U.S
Source: PLoS One. 2025 Aug 26;20(8):e0315259. doi: 10.1371/journal.pone.0315259 (PMC12380328; doi:10.1371/journal.pone.0315259)
Supplement: S3 Appendix — (DOCX) [file pone.0315259.s003.docx]

**Appendix 3: Factor Analysis Results**

The Need for Cognition scale (Maksl et al, 2015) was subjected to exploratory factor analysis (EFA), using SPSS version 29. Because the author’s experience with the scale had been confined to small samples, it was decided to use EFA instead of CFA (confirmatory factor analysis) for the large samples collected for this study.

The EFA revealed the five items loaded on two factors, indicating two sub-scales. The three items that loaded on the first sub-scale were MAC1R, MAC2R, and MAC5R. The other two items (MAC3 and MAC4) loaded on the second sub-scale. [The items are reproduced here for convenience: (MAC1) I don’t like to have to do a lot of thinking; (MAC2) I try to avoid situations that require thinking in depth about something: (MAC3) I prefer to do something that challenges my thinking abilities rather than something that requires little thought; (MAC4) I prefer complex to simple problems; (MAC5) Thinking hard and for a long time about something gives me little satisfaction.] Items 1, 2 and 5 were reverse coded and needed to be recalculated for analysis. The table below provides the results of the analyses.

| **Items** | **Loadings** | **AVE** | **CR** | **Cronbach’s alpha** |
| --- | --- | --- | --- | --- |
| MAC1R | 0.858 |  |  |  |
| MAC2R | 0.86 |  |  |  |
| MAC5R | 0.679 |  |  |  |
|  |  | 0.646 | 0.844 | 0.721 |
| MAC3 | 0.28 |  |  |  |
| MAC4 | 0.117 |  |  |  |
|  |  | 0.007 | 0.1 | 0.653 |

According to Hair et al (2019), factor loadings of +/- 0.5 are “considered practically significant,” while those exceeding +/- 0.7 are considered “indicative of well-defined structure” (p. 151). The loading for MAC5R is just less than 0.7, indicating good loadings for the first sub-scale. Also, Hair et al (2019) state that the lower limit for Cronbach’s alpha is 0.7 (p. 161), AVE estimates should exceed 0.5, and construct reliability should be 0.7 or better (p. 663). The three-item scale for Need for Cognition, based on MAC1R, MAC2R and MAC5R, is psychometrically strong.

With a model with only three indicators, the model is saturated, with no degrees of freedom, so it was not possible to generate goodness of fit measures using SEM for a CFA.

I then created a summated scale for Need for Cognition based on the three-item scale by summing the values of the items and dividing them by three.

For this version of the paper, the editor asked that MAC5R be removed from the scale. Without MAC5R, Cronbach’s alpha is .863; CR is .849; AVE is .738. All three values exceed those for the original three-item scale. I then created a summated scale based on the two-item scale by summing the values of the items and dividing them by two. This revised scale was used for all analyses in the current version of the paper.

References:

Hair, J.F. Jr., Black, W.C., Babin, B.J. and Anderson, R.E. *Multivariate Data Analysis* (8e) 2019. Andover, Hampshire, UK: Cengage.

Maksl, A., Ashley, S., & Craft, S. Measuring news media literacy. *Journal of Media Literacy Education* 2015; 6(3): 29-45.
