# Supplementary material for: Political affiliation or need for cognition? It depends on the post: Comparing key factors related to detecting health disinformation in the U.S
Source: PLoS One. 2025 Aug 26;20(8):e0315259. doi: 10.1371/journal.pone.0315259 (PMC12380328; doi:10.1371/journal.pone.0315259)
Supplement: S4 Appendix — (DOCX) [file pone.0315259.s004.docx]

**Appendix 4: Statistical Test Results for Non-Significant Posts**

**Questionnaire Version 1**

**Omnibus Test**

| **Question** | **Likelihood Ratio Chi-Square** | **df** | **Significance** |
| --- | --- | --- | --- |
| 2: Semaglutide | 4.747 | 3 | .191 |
| 5: Antibiotics & viruses | 6.801 | 3 | .079 |
| 6: Pro-Chlorine Dioxide | 6.440 | 3 | .092 |
| 7: Pro-MMR vaccine | 1.321 | 3 | .724 |
| 8: Muscle supplements | 6.270 | 3 | .099 |

**Tests of Model Effects**

Model: (Intercept), Political Affiliation, Need for Cognition; Dependent Variable: Correct judgment

| **Question** | **Variable** | **Wald Chi-Square** | **df** | **Significance** |
| --- | --- | --- | --- | --- |
| 2: Semaglutide | (Intercept) | .450 | 1 | .502 |
|  | Political Affiliation | 2.630 | 2 | .269 |
|  | Need for Cognition | 1.759 | 1 | .1.85 |
| 5: Antibiotics & viruses | (Intercept) | 93.980 | 1 | <.001 |
|  | Political Affiliation | 5.840 | 2 | .054 |
|  | Need for Cognition | .005 | 1 | .946 |
| 6: Pro-Chlorine Dioxide | (Intercept) | 58.812 | 1 | <.001 |
|  | Political Affiliation | .451 | 2 | .778 |
|  | Need for Cognition | 5.889 | 1 | .015 |
| 7: Pro-MMR vaccine | (Intercept) | 1.390 | 1 | .238 |
|  | Political Affiliation | 1.311 | 2 | .519 |
|  | Need for Cognition | .019 | 1 | .890 |
| 8: Muscle supplements | (Intercept) | 61.045 | 1 | <.001 |
|  | Political Affiliation | 2.752 | 2 | .253 |
|  | Need for Cognition | 4.276 | 1 | .039 |

**Questionnaire Version 2**

**Omnibus Test**

| **Question** | **Likelihood Ratio Chi-Square** | **df** | **Significance** |
| --- | --- | --- | --- |
| 2: Intermittent fasting | 0.071 | 3 | .985 |
| 3: Nyquil | 1.386 | 3 | .709 |
| 7: FDA chlorine dioxide | 3.896 | 3 | .308 |
| 9: Harvard Public Health | 3.155 | 3 | .368 |
| 10: Lysulin | 7.142 | 3 | .068 |

**Tests of Model Effects**

Model: (Intercept), Political Affiliation, Need for Cognition; Dependent Variable: Correct judgment

| **Question** | **Variable** | **Wald Chi-Square** | **df** | **Significance** |
| --- | --- | --- | --- | --- |
| 2: Intermittent fasting | (Intercept) | 4.539 | 1 | .033 |
|  | Political Affiliation | .053 | 2 | .974 |
|  | Need for Cognition | .019 | 1 | .890 |
| 3: Nyquil | (Intercept) | 98.723 | 1 | <.001 |
|  | Political Affiliation | 1.307 | 2 | .520 |
|  | Need for Cognition | .112 | 1 | .738 |
| 7: FDA chlorine dioxide | (Intercept) | 86.843 | 1 | <.001 |
|  | Political Affiliation | 2.750 | 2 | .253 |
|  | Need for Cognition | .687 | 1 | .407 |
| 9: Harvard Public Health | (Intercept) | 20.890 | 1 | <.001 |
|  | Political Affiliation | 3.030 | 2 | .220 |
|  | Need for Cognition | .064 | 1 | .800 |
| 10: Lysulin | (Intercept) | 38.680 | 1 | <.001 |
|  | Political Affiliation | 2.228 | 2 | .328 |
|  | Need for Cognition | 4.773 | 1 | .029 |
